# Differential distribution of manifest lesions in diabetic retinopathy by fundus fluorescein angiography and fundus photography

**DOI:** 10.1186/s12886-020-01740-2

**Published:** 2020-12-01

**Authors:** Xiaoli Li, Jie Xie, Liang Zhang, Ying Cui, Guanrong Zhang, Jun Wang, Aiping Zhang, Xiangting Chen, Tian Huang, Qianli Meng

**Affiliations:** 1 Department of Ophthalmology, Guangdong Provincial People’s Hospital, Guangdong Academy of Medical Sciences, Guangdong Eye Institute, 106 Zhongshan Er Road, Guangzhou, 510080 People’s Republic of China; 2 State Key Laboratory of Ophthalmology, Zhongshan Ophthalmic Center, Sun Yat-sen University, Guangzhou, China; 3 Information and Statistical Center, Guangdong Provincial People’s Hospital, Guangdong Academy of Medical Sciences, Guangzhou, Guangdong China; 4 The Second School of Clinical Medicine, Southern Medical University, Guangzhou, China

**Keywords:** Manifest lesions, Diabetic retinopathy, Retinal-imaging, Distribution

## Abstract

**Background:**

To analyze the distribution of manifest lesions of diabetic retinopathy (DR) by fundus fluorescein angiography (FFA) and color fundus photography (FP).

**Methods:**

A total of 566 eyes of 324 Chinese patients diagnosed with DR were included in this retrospective study. DR severity was graded by the international grading criterion. The distributions of microaneurysms (MA), intraretinal hemorrhages/exudates (He/Ex), intraretinal microvascular abnormality (IRMA), capillary nonperfusion areas (NPA), and neovascularization (NV) were estimated by multiple logistic regression analyse based on nine-field FFA and FP images.

**Results:**

In mild nonproliferative diabetic retinopathy (NPDR), the highest frequency of MA was found in the posterior pole (67.7%), followed by the inferior nasal (59.4%), and the nasal (55.4%) fields. In moderate NPDR, MA frequently distributed in the posterior pole (98.0%), nasal (97.0%), superior (96.0%), inferior nasal (94.9%), and inferior (92.9%) fields, whereas He/Ex were most prevalent in the posterior pole (69.7%). In severe NPDR and proliferative DR, IRMA, NPA, and NV were more frequent in the nasal field, particularly in the inferior nasal field (60.3, 38.7, and 76.0%, respectively). All lesions were more observed in the combined posterior pole, nasal, and inferior nasal fields than in the posterior pole or combined two fields in the early and severe stages of DR (*P* < 0.05).

**Conclusions:**

The manifest lesions of DR were common in the nasal field besides the posterior pole in Chinese patients. A combined examination of the posterior pole, nasal, and inferior nasal mid-peripheral retina would help to detect different retinal lesions of DR.

**Trial registration:**

ClinicalTrial. gov, NCT03528720. Registered 18 May 2018 - Retrospectively registered.

## Background

Diabetic retinopathy (DR) is characterized by structural and functional alterations in the retinal microvasculature [1], which causes capillary occlusion, vascular hyperpermeability, and neovascularization (NV) in the retina [2]. Previous studies have found that vascular lesions in DR are not distributed uniformly within the retina [3–7], but the results are quite different. In the early stages of non-proliferative diabetic retinopathy (NPDR), microaneurysms (MA) are found to locate most commonly in the posterior pole lateral to the macula [8] or in the temporal field [9]. As the disease progresses, the intraretinal microvascular abnormality (IRMA) and capillary nonperfusion areas (NPA) are reported to involve the mid-peripheral retina and the larger vascular arcades [6, 7, 10, 11] or appear in the nasal field [12, 13]. In proliferative diabetic retinopathy (PDR), Feman et al. found that neovascularization elsewhere in the retina (NVE) arose most frequently in the superotemporal quadrant at 6 mm from the optic disk [14], whereas Jansson et al. showed that the majority of NVE lesions are located inferonasal to the optic disc and along the superior vascular arcades [1]. Considering these discordant findings, therefore, it is necessary to identify the distribution characteristics of the DR lesions in different populations of diabetic patients, which may help to clarify predisposition to or protection against certain areas of diabetes-induced retinal changes and improve strategies for screening and diagnosis of DR.

Early Treatment of Diabetic Retinopathy Study (ETDRS) 7 stand-field 35-mm mydriatic stereoscopic 30-degree color fundus photography has been widely considered the gold standard for evaluation of DR in both clinical and research settings [9, 15]. The assessment of DR severity by using ETDRS photographs relies only on retinal lesions located within this posterior area of the retina and does not define criteria for retinal lesions located outside the imaged area [9]. With the advances of retinal imaging technology, 1-field, 3-field, multi-field, or ultra-wide field digital color imaging is reported to be used for DR grading and screening, but the results are not consistent in literature [6, 9, 16].

To clarify the regional distribution of the manifest lesions of DR, as well as to explore the disease-sensitive areas for diagnosis and monitoring of DR, we retrospectively analyzed the distribution of MA, intraretinal hemorrhages/exudates (He/Ex), IRMA, NPA, and NV of differently graded DR in nine-field fundus fluorescein angiography (FFA) and color fundus photography (FP) images in this study.

## Methods

### Patients

The present study was a hospital-based, retrospective study. All 55-degree FFA (Spectralis HRA, Heidelberg Engineering, Germany) and 45-degree color FP (TRC-NW8, TOPCON, Japan) images from patients with type 1 or type 2 diabetes mellitus who were diagnosed with DR by comprehensive ophthalmic examination from January 2014 to December 2016, were included in this study. Exclusion criteria included poor image quality due to refractive opacity or misalignment, any fundus disease except for DR, and history of ophthalmological intervention procedure (e.g., laser photocoagulation, vitrectomy, anti-VEGF injection in one or both eyes). This study complied with the Declaration of Helsinki for research involving human subjects and was approved by the research ethics committee of Guangdong Provincial People’s Hospital.

### Definitions of DR stages and fundus image fields

**Fig. 1:**
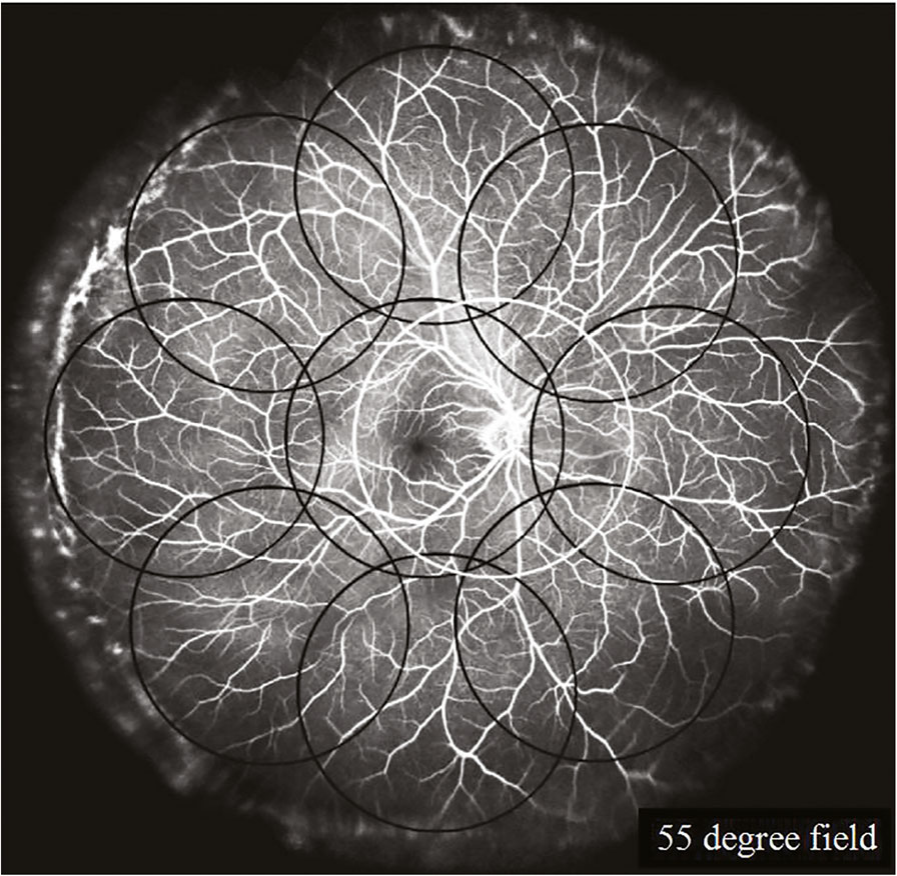
The FFA, Spectralis HRA image. The field of view is 55-degrees and captures the image in nine black circles, respectively [17]

Although ETDRS severity scale is recognized as the “gold standard” for grading the severity of DR in clinical trials, its use in everyday clinical practice has not proven to be easy or practical [17]. In recent more than 10 years, the International Clinical Diabetic Retinopathy Disease Severity Scale provides an important basis for standardizing DR and it is widely used in worldwide. Therefore, based on the Diabetic Retinopathy Disease Severity Scale in this study, DR was classified as follows: no diabetic retinopathy (NDR, no abnormalities), mild NPDR (only subjects with MA), moderate NPDR (subjects with more than just MA but less than severe NPDR), severe NPDR (subjects with any of the following: more than 20 intraretinal hemorrhages in each of 4 quadrants; definite venous beading [VB] in 2+ quadrants; prominent IRMA in 1+ quadrant; and no signs of PDR), and PDR (subjects with one or more of the following: NVE, neovascularization of the disc (NVD), vitreous/preretinal hemorrhage) [17]. In order to show the retinal lesions from the posterior pole to the peripheral retina as clearly as possible, the mydriatic images of FFA and FP were divided into nine fields and were centered on the following areas: the macula (posterior pole), superior mid-peripheral retina, superior temporal mid-peripheral retina, temporal mid-peripheral retina, inferior temporal mid-peripheral retina, inferior mid-peripheral retina, inferior nasal mid-peripheral retina, nasal mid-peripheral retina, and superior nasal mid-peripheral retina (Fig. 1) [18, 19]. The posterior pole was defined as 1 disc diameter (DD) away from the superior, nasal, inferior side to the disc and 1 to 4 DD away from the temporal side of the disc, which consisted of the optic disc, macula, and temporal vessel arches. The mid-peripheral retina was defined as 1 to 4 DD away from the nasal side of the disc and 4 to 6 DD away from the temporal side of the disc. The disease severity level and distribution of typical clinical signs of DR, including MA, intraretinal hemorrhages, exudates (hard exudates and cotton-wool spots), VB, IRMA, NPA, NVE, and NVD, were graded and evaluated based on FFA and FP images by two ophthalmologists (J. W. and X. L.). In cases of disagreement, consensus was reached by discussion with a third ophthalmologist (Q.M.). It is worth noting that the angle of some images might be shown a small deviation due to patient’s poor cooperation during FFA. However, the whole mid-peripheral retina could be clearly displayed because of the overlap of images in each field, according to which the clinical signs of DR could still be accurately identified.

### Statistics

Statistical analyses were performed with SPSS 23.0 software (SPSS. Inc., Chicago, IL, USA). Both qualitative and quantitative variables were presented as number and percentage. Differences in the frequency of clinical signs in nine fields in different DR stages were estimated by multiple logistic regression analyses. Odds ratios and 95% confidence intervals were presented. Significant differences were defined as *P* < 0.05.

## Results

### Clinical characteristics of patients

A total of 566 eyes of 324 patients with DR were enrolled in the study, and their demographic and clinical characteristics are shown in Table 1. The mean age was 57.2 ± 10.9 years (24–84 years), and 57.4% of patients were male (186 persons, 316 eyes). Sixty-five eyes had mild NPDR (11.5%), 99 eyes had moderate NPDR (17.5%), 194 eyes had severe NPDR (34.3%), and 208 eyes had PDR (36.8%).

**Table 1:** Demographic and clinical characteristics of patients included in the study

	Total	Mild NPDR	Moderate NPDR	Severe NPDR	PDR
N (eyes)	566	65 (11.5%)	99 (17.5%)	194 (34.3%)	208 (36.8%)
Age (yrs, mean ± SD)	57.2 ± 10.9	59.8 ± 11.2	58.0 ± 11.6	58.3 ± 11.4	55.1 ± 9.7
Male (eyes)	316 (55.8%)	47 (72.3%)	51 (51.5%)	107 (55.2%)	111 (53.4%)
Female (eyes)	250 (44.2%)	18 (27.7%)	48 (48.5%)	87 (44.9%)	97 (46.6%)

*NPDR* nonproliferative diabetic retinopathy, *PDR* proliferative diabetic retinopathy

### Distribution of MA in mild NPDR

As the earliest clinically recognizable lesion in DR, the distribution of MA in the nine fields exhibited a statistically significant difference (F = 6.39, *P* < 0.001). The highest frequency of MA were observed in the posterior pole (67.7%), followed by the inferior nasal (59.4%) and the nasal fields (55.4%), without statistically significant differences among the three fields (Table 2).

**Table 2:** The frequency of microaneurysms in the nine fields in mild nonproliferative diabetic retinopathy (*n* = 65 eyes)

Field	N (%)	OR (95% CI)	*P*
Posterior pole	44 (67.7)	reference	
Superior	33 (50.8)	0.34 (0.14–0.82)	0.017
Superior temporal	21 (32.3)	0.09 (0.03–0.23)	< 0.001
Temporal	23 (35.4)	0.11 (0.04–0.28)	< 0.001
Inferior temporal	19 (29.2)	0.07 (0.03–0.18)	< 0.001
Inferior	28 (43.1)	0.20 (0.08–0.49)	0.001
Inferior nasal	38 (59.4)	0.58 (0.24–1.38)	0.216
Nasal	36 (55.4)	0.46 (0.19–1.10)	0.080
Superior nasal	30 (46.2)	0.25 (0.10–0.61)	0.002

*OR* odds ratios, *CI* confidence interval

### Distributions of MA and He/Ex in moderate NPDR

Because intraretinal hemorrhages and exudates are the other important fundus lesions for moderate NPDR, they were recorded as one clinical sign in this DR stage. There were statistically significant differences for the frequencies of MA (F = 14.5, *P* < 0.01) and He/Ex (F = 19.54, *P* < 0.001) in the nine fields. MA were more prevalent in the posterior pole (98.0%), nasal (97.0%), superior (96.0%), inferior nasal (94.9%), and inferior (92.9%) retina, whereas He/Ex were most frequently found in the posterior pole (69.7%) but were significantly lower in other fields (Table 3).

**Table 3:** The frequencies of microaneurysms and intraretinal hemorrhages/exudation in the nine fields in moderate nonproliferative diabetic retinopathy (*n* = 99 eyes)

Field	MA			He/Ex		
	N (%)	OR (95% CI)	*P*	N (%)	OR (95% CI)	*P*
Posterior pole	97 (98.0)	reference		69 (69.7)	reference	
Superior	95 (96.0)	0.42 (0.06–2.78)	0.367	23 (23.2)	0.10 (0.05–0.20)	< 0.001
Superior temporal	67 (67.7)	0.01 (0.003–0.07)	< 0.001	4 (4.0)	0.01 (0.004–0.04)	< 0.001
Temporal	61 (61.6)	0.01 (0.002–0.05)	< 0.001	5 (5.1)	0.02 (0.01–0.04)	< 0.001
Inferior temporal	64 (64.6)	0.01 (0.002–0.06)	< 0.001	4 (4.0)	0.01 (0.004–0.04)	< 0.001
Inferior	92 (92.9)	0.19 (0.03–1.13)	0.067	13 (13.1)	0.05 (0.02–0.10)	< 0.001
Inferior nasal	94 (94.9)	0.31 (0.05–1.95)	0.210	13 (13.1)	0.05 (0.02–0.10)	< 0.001
Nasal	96 (97.0)	0.61 (0.08–4.41)	0.622	16 (16.2)	0.06 (0.03–0.13)	< 0.001
Superior nasal	89 (89.9)	0.11 (0.02–0.62)	0.013	16 (16.2)	0.06 (0.03–0.13)	< 0.001

*MA* microaneurysms, *He/Ex* intraretinal hemorrhages/exudation, *OR* odds ratios, *CI* confidence interval

### Distributions of IRMA and NPA in severe NPDR

In severe NPDR, besides intraretinal hemorrhages, IRMA, VB, and NPA were the other important clinical signs that were clearly shown in FFA images. Intraretinal hemorrhages were usually widespread throughout the retina, while VB was rarely observed in Chinese patients (frequency of VB was 5.9% [31/526 eyes] in Chen’s report [19] and 6.2% [12/194 eyes] in our study) and thus it was not analyzed in this study. Therefore, the distributions of IRMA and NPA were analyzed as typical lesions in this DR stage. As shown in Table 4, the frequencies of IRMA (F = 34.68, *P* < 0.001) and NPA (F = 18.77, *P* < 0.001) were significantly different among the nine fields. The highest frequency of IRMA (60.3%) was found in the inferior nasal mid-peripheral retina with a statistically significant difference compared with the posterior pole, followed by the nasal (53.6%), superior nasal (52.1%), inferior (49.0%), posterior pole (49.0%), and superior retina (41.8%). NPA were most frequently found in 38.7% of the inferior nasal retina, 29.9% of the superior nasal, and 29.4% of the nasal retina, which were significantly higher compared with that in the posterior pole (17.0%).

**Table 4:** The frequencies of intraretinal microvascular abnormality and capillary nonperfusion areas in the nine fields in severe nonproliferative diabetic retinopathy (*n* = 194 eyes)

Field	IRMA			NPA		
	N (%)	OR (95% CI)	*P*	N (%)	OR (95% CI)	*P*
Posterior pole	95 (49.0)	reference		33 (17.0)	reference	
Superior	81 (41.8)	0.68 (0.43–1.08)	0.102	36 (18.6)	1.14 (0.63–2.06)	0.653
Superior temporal	21 (10.8)	0.07 (0.04–0.13)	< 0.001	6 (3.1)	0.11 (0.04–0.29)	< 0.001
Temporal	18 (9.3)	0.06 (0.03–0.11)	< 0.001	8 (4.1)	0.15 (0.06–0.37)	< 0.001
Inferior temporal	15 (7.7)	0.04 (0.02–0.09)	< 0.001	13 (6.7)	0.28 (0.13–0.58)	0.001
Inferior	95 (49.0)	1.00 (0.63–1.58)	1.00	38 (19.6)	1.25 (0.70–2.24)	0.458
Inferior nasal	117 (60.3)	1.82 (1.15–2.87)	0.011	75 (38.7)	4.37 (2.54–7.52)	< 0.001
Nasal	104 (53.6)	1.27 (0.81–2.01)	0.297	57 (29.4)	2.52 (1.44–4.38)	0.001
Superior nasal	101 (52.1)	1.18 (0.75–1.85)	0.486	58 (29.9)	2.60 (1.49–4.52)	0.001

*IRMA* intraretinal microvascular abnormality, *NPA* capillary nonperfusion areas, *OR* odds ratios, *CI* confidence interval

### Distributions of NV in PDR

In view of NVE and NVD as the representative clinical manifestations of PDR, NV of the optic disc or in the retina was analyzed. There was a statistically significant difference in the frequency of NV among the nine fields (F = 42.81, *P* < 0.001). The frequency of NV in the inferior nasal field was the highest (76.0%), with a statistically significant difference compared to the posterior pole (including NVD, 63.5%). In the superior, inferior, nasal, and superior nasal retina, it was similar to that in the posterior pole (Table 5).

**Table 5:** The frequency of neovascularization in the nine fields in proliferative diabetic retinopathy (*n* = 208 eyes)

Field	N (%)	OR (95% CI)	*P*
Posterior pole	132 (63.5)	reference	
Superior	144 (69.2)	1.36 (0.87–2.12)	0.175
Superior temporal	51 (24.5)	0.13 (0.08–0.21)	< 0.001
Temporal	49 (23.6)	0.12 (0.08–0.20)	< 0.001
Inferior temporal	48 (23.1)	0.12 (0.08–0.19)	< 0.001
Inferior	121 (58.2)	0.77 (0.50–1.18)	0.226
Inferior nasal	158 (76.0)	2.02 (1.28–3.21)	0.003
Nasal	145 (69.7)	1.40 (0.89–2.18)	0.141
Superior nasal	146 (70.2)	1.44 (0.92–2.25)	0.112

*OR* odds ratios, *CI* confidence interval

### Frequencies of clinical signs in combined fields

**Fig. 2:**
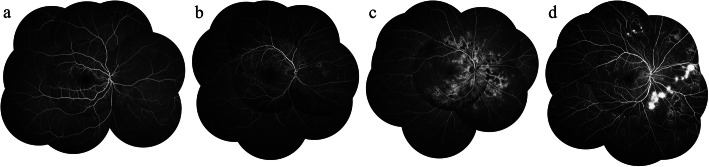
The typical FFA images of different diabetic retinopathy stages. **a** mild NPDR; **b** moderate NPDR; **c** severe NPDR; **d** PDR. NPDR: nonproliferative diabetic retinopathy; PDR: proliferative diabetic retinopathy

**Fig. 3:**
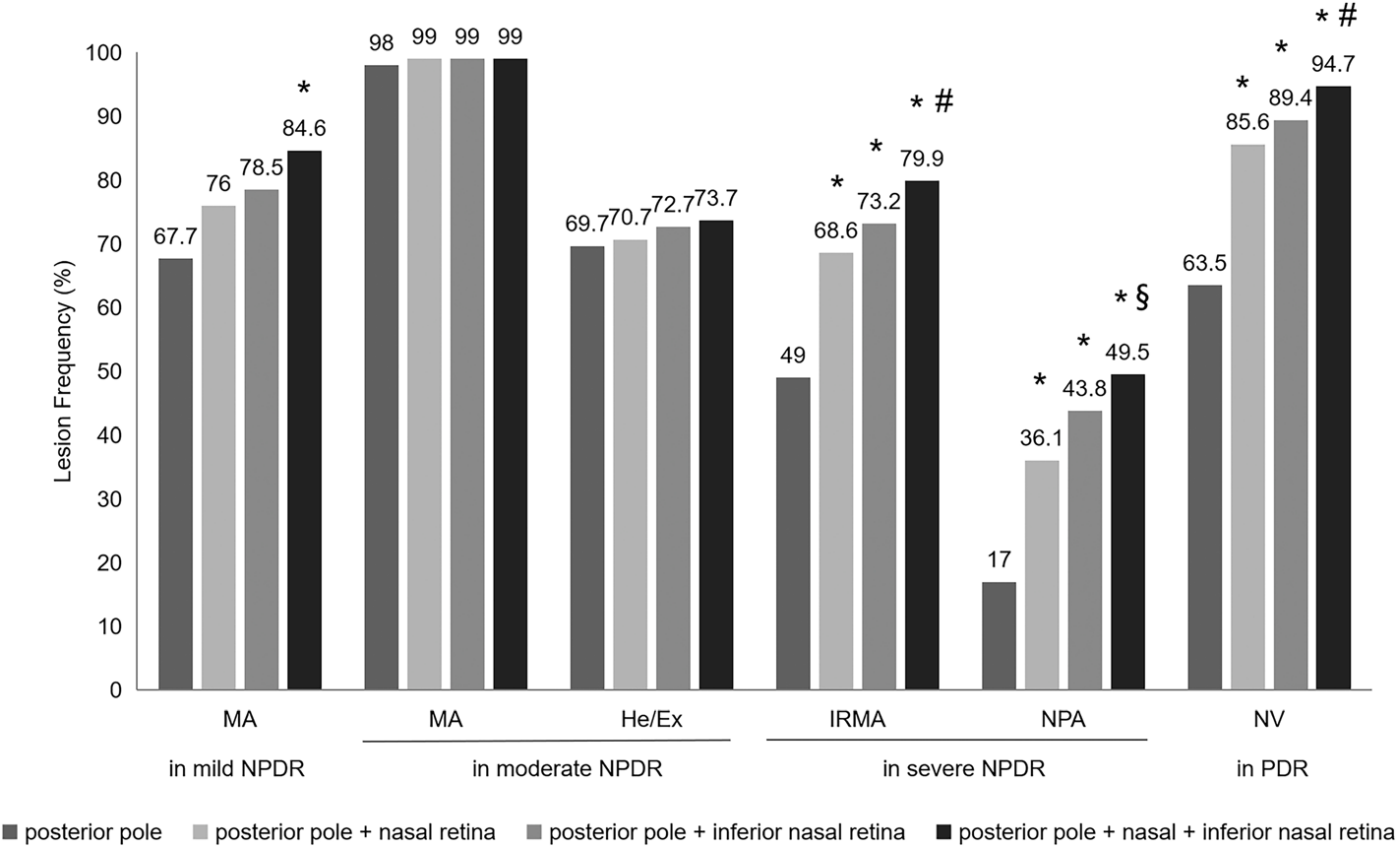
The frequencies of clinical signs of diabetic retinopathy in the posterior pole, the combined two and three fields. MA: microaneurysms; He/Ex: intraretinal hemorrhages and exudates; IRMA: intraretinal microvascular abnormality; NPA: capillary nonperfusion area; NV: neovascularization; NPDR: nonproliferative diabetic retinopathy; PDR: proliferative diabetic retinopathy. *Compared to the posterior pole, *P* < 0.05. #Compared to two combined fields respectively, *P* < 0.05. §Compared to combined field with the posterior pole and the nasal retina, *P* < 0.05

A combined examination of two or more fields usually improves the rate of DR detection. Based on the preceding results, we found that these clinical signs of DR were more prevalent in the posterior pole, nasal, and inferior nasal retina (Table 6, Fig. 2). Therefore, we further compared their frequencies in the posterior pole, posterior pole combined with the nasal field, posterior pole combined with the inferior nasal field, and posterior pole combined with the nasal and inferior nasal field, respectively. As shown in Fig. 3, the frequency of MA in mild NPDR in the three fields (posterior pole + nasal + inferior nasal) was 84.6%, significantly higher than that in the posterior pole (67.7%; *P* = 0.002), but similar to those in the two fields (posterior pole + nasal: 76.9%, posterior pole + inferior nasal: 78.5%), respectively. In moderate NPDR, there was no statistically significant difference in frequencies of MA (F = 0.31, *P* = 0.816) and He/Ex (F = 0.50, *P* = 0.680) among the posterior pole, combined two fields, and three fields. In severe NPDR and PDR, a similar distribution trend was observed for IRMA, NPA, and NV, that their frequencies in the combined three fields were significantly higher than those in the posterior pole and the combined two fields (posterior pole + nasal, or posterior pole + inferior nasal), respectively.

**Table 6:** The distribution of clinical signs at different diabetic retinopathy stages^*a*^

Stages	Posterior pole	Superior	Superior temporal	Temporal	Inferior temporal	Inferior	Inferior nasal	Nasal	Superior nasal
Mild NPDR	MA	–	–	–	–	–	MA	MA	–
Moderate NPDR	MA He/Ex	MA	–	–	–	MA	MA	MA	–
Severe NPDR	IRMA NPA	IRMA NPA	–	–	–	IRMA NPA	IRMA NPA	IRMA NPA	IRMA NPA
PDR	NV	NV	–	–	–	NV	NV	NV	NV

^*a*^Fields in which the frequencies of clinical signs are significantly higher than or similar to those of the posterior pole are shown in this table

*MA* microaneurysms, *He/Ex* intraretinal hemorrhages/exudation, *IRMA* intraretinal microvascular abnormality, *NPA* capillary nonperfusion areas, *NV* neovascularization, *NPDR* nonproliferative diabetic retinopathy, *PDR* proliferative diabetic retinopathy

## Discussion

The present study retrospectively analyzed the distribution of manifest lesions of different DR stages in nine fields of FFA and FP images in patients with DR. Our results showed that MA was early and frequently occurred in the posterior pole centered on the macula, followed by the nasal and nasal inferior mid-peripheral retina. As the disease progressed, He/Ex was located more in the posterior pole, whereas IRMA, NPA, and NV were frequently observed in the nasal field (including nasal, inferior nasal, and superior nasal mid-peripheral retina). The imaging in the posterior pole combined with the nasal and inferior nasal retina showed more lesions, suggesting that using it would improve the rate of DR detection and assessment of the DR severity in a screening setting.

There is evidence for a geographic variability in the distribution of manifest lesions in DR [3–7]. Except most often at the posterior pole of the retina, MA, IRMA, and NPA were also reported to be more prevalent in the superior temporal [20], nasal [4, 12, 13], temporal [9], or mid-peripheral [10, 21] retina on the basis of experimental work in diabetic animals and human autopsy specimens, or by using ETDRS 7-standard 30-degree stereoscopic color fundus photographs, or 45- or 55-degree digital color retinal images, or ultra-wide field imaging technologies. The possible reasons for the different results may be related to the different regional standards and retinal imaging technology. ETDRS 7-standard 30-degree fields, four quadrants (nasal, temporal, superior and inferior retina) or two quadrants (nasal and temporal hemisphere) centered on the optic disc, were used in previous studies. ETDRS 7-standard fields cover approximately the central posterior 90 degrees of the retina, representing only approximately 30% of the entire retinal surface area [9]. However, the nine 55-degree fields centered on the macula used in our study covered approximately the 150 degrees of the retina by combination images, representing approximately 60% of the retinal area, implying that more lesions could be located and described accurately and clearly by this technology. Of course, it has a relatively higher requirement for patient cooperation than that in ultra-wide field (UWF) imaging technology. Although an UWF 200-degree imaging technology, representing approximately 82% of the retinal area, will be available for single-image analysis of the fundus periphery in the future, this technology has several details that should be improved. One recent study showed that the temporal quadrant had the widest distribution of predominantly peripheral lesions using UWF fundus imaging [22], which different from our results that the manifest lesions were common in the mid-peripheral nasal field besides the posterior pole using FFA imaging with nine 55-degree fields. In view of the different retinal region observed using different imaging technology in the two articles, the different results shown were not contradictory in fact. We speculate that in addition to the posterior pole, the manifest lesions of DR may often distribute in the mid-peripheral nasal and peripheral temporal retina according to these results.

Our study showed that DR displayed regional differences, with the formation of MA, hemorrhages, and exudates related to hyperperfusion in the posterior pole, as well as with the formation of IRMA, NPA, and NV related to capillary occlusion and retinal ischemia in the nasal mid-peripheral retinal areas. The distribution of retinal capillaries and retinal thickness has been reported as one of the possible reasons. There are 4 layers of capillary network in the radial peripapillary capillary and 3 layers in the macula, and MA and He/Ex tend to present in these blood flow–rich areas. The retinal capillary network drops to 2 layers in the mid-periphery and to 1 layer at the ora serrata of the periphery. The blood supply of the mid-periphery is worse than that of the posterior pole, and the retina is weak in the thinning of the capillary network, which is prone to ischemia, showing as IRMA and NPA [23, 24]. Meanwhile, Skov Jensen et al. speculated that these regional differences in the occurrence of retinopathy lesions might reflect differences in the capacity of retinal arterioles to autoregulate blood flow by adapting the diameter of retinal arterioles [25]. In the central retinal areas, the diameter response to increased blood pressure and retinal metabolism interacted in a way that could potentially protect this area from ischemia, whereas this protective mechanism was absent in the peripheral retinal arterioles [25]. In addition, hemoglobin oxygen saturation of retinal arterioles and venules has been demonstrated to increase with DR severity [26], suggesting that retinal oxygen saturation may be associated with the distribution of DR lesions. Other possible reasons include the difference of race, disease condition, and detection technique. Since currently available hypotheses fail to account completely for regional differences in the distribution of the vascular lesions within the same retina, the exact mechanism remains to be confirmed by further study.

It is very important for diabetic patients to undergo regular ocular examination, but fewer than half of them receive recommended screening [27]. In the present study, the frequencies of the DR lesions in the posterior pole combined with the nasal and inferior nasal mid-peripheral retina were significantly higher than those in the posterior pole or in the two combined fields, especially in the early or severe stages of DR, suggesting that the examination of the three retinal fields may be an effective method in a screening setting to detect different retinal lesions of DR for providing advice about treatment and follow-up. Further study is needed to evaluate the sensitivity and specificity of the three-field images for DR diagnosis and grading.

Several limitations to our study need to be considered. First, as a hospital-based retrospective study, selection bias may have occurred when patients were recruited into this study. Second, there might have been some subjectivity in reading FFA and FP images and counting the clinical signs. Third, peripheral retinopathy is difficult to observe by nine-field 55-degree images due to the limitation of the machine and technique. In addition, as a complication of diabetes mellitus, it is worth while to further study the relationship between patient’s medical problem and clinical signs of DR.

## Conclusions

Our study demonstrated that the typical clinical signs of DR, including MA, IRMA, NPA, and NV, were more common in the nasal and inferior nasal mid-peripheral retina in addition to the posterior pole. A combined examination of the posterior pole, nasal, and inferior nasal mid-peripheral retina may help to improve the detection of different retinal lesions of DR in a screening setting when the ultra-wide field imaging technology is unavailable. Future prospective trials will be performed to determine whether the mid-peripheral changes in particular fields are associated with risk of DR progression.

## Data Availability

The data used in the current study is available from the corresponding author upon request.

## Abbreviations

DD: Disc diameter
DR: Diabetic retinopathy
ETDRS: Early Treatment of Diabetic Retinopathy Study
FFA: Fundus fluorescein angiography
FP: Color fundus photography
He/Ex: Intraretinal hemorrhages/exudates
IRMA: Intraretinal microvascular abnormality
MA: Microaneurysms
NPA: Capillary nonperfusion areas
NPDR: Nonproliferative diabetic retinopathy
NV: Neovascularization
NVD: Neovascularization of the disc
NVE: Neovascularization elsewhere in the retina
PDR: Proliferative diabetic retinopathy
VB: Venous beading
